# Rapid nanobody-based imaging of mesothelin expressing malignancies compatible with blocking therapeutic antibodies

**DOI:** 10.3389/fimmu.2023.1200652

**Published:** 2023-06-14

**Authors:** Abdennour Benloucif, Damien Meyer, Laure Balasse, Armelle Goubard, Lucile Danner, Ahlem Bouhlel, Rémy Castellano, Benjamin Guillet, Patrick Chames, Brigitte Kerfelec

**Affiliations:** ^1^ Aix Marseille Univ, CNRS, INSERM, Institut Paoli-Calmettes, CRCM, Marseille, France; ^2^ Aix Marseille Univ, CNRS, Centre Européen de Recherche en Imagerie Medicale (CERIMED), Marseille, France; ^3^ Aix-marseille University, INSERM, INRAE, Centre de recherche en Cardiovasculaire et Nutrition (C2VN), Marseille, France; ^4^ Aix Marseille Univ, CNRS, INSERM, Institut Paoli-Calmettes, CRCM, TrGET Preclinical Platform, Marseille, France

**Keywords:** mesothelin (MSLN), nanobodies (Nbs), positron emission tomography - computed tomography, fluorescence imaging, diagnostic, site-directed conjugation

## Abstract

**Introduction:**

Mesothelin (MSLN) is overexpressed in a wide variety of cancers with few therapeutic options and has recently emerged as an attractive target for cancer therapy, with a large number of approaches currently under preclinical and clinical investigation. In this respect, developing mesothelin specific tracers as molecular companion tools for predicting patient eligibility, monitoring then response to mesothelin-targeting therapies, and tracking the evolution of the disease or for real-time visualisation of tumours during surgery is of growing importance.

**Methods:**

We generated by phage display a nanobody (Nb S1) and used enzymatic approaches were used to site-directed conjugate Nb S1 with either ATTO 647N fluorochrome or NODAGA chelator for fluorescence and positron emission tomography imaging (PET) respectively.

**Results:**

We demonstrated that Nb S1 displays a high apparent affinity and specificity for human mesothelin and demonstrated that the binding, although located in the membrane distal domain of mesothelin, is not impeded by the presence of MUC16, the only known ligand of mesothelin, nor by the therapeutic antibody amatuximab. *In vivo* experiments showed that both ATTO 647N and [^68^Ga]Ga-NODAGA-S1 rapidly and specifically accumulated in mesothelin positive tumours compared to mesothelin negative tumours or irrelevant Nb with a high tumour/background ratio. The *ex vivo* biodistribution profile analysis also confirmed a significantly higher uptake of Nb S1 in MSLN-positive tumours than in MSLN^low^ tumours.

**Conclusion:**

We demonstrated for the first time the use of an anti-MSLN nanobody as PET radiotracer for same day imaging of MSLN^+^ tumours, targeting an epitope compatible with the monitoring of amatuximab-based therapies and current SS1-derived-drug conjugates.

## Introduction

An increasingly detailed understanding of the tumour process and the development of cutting-edge technologies and approaches are leading to major strategic changes in cancer treatment modalities, with a strong orientation towards combinatorial strategies and adaptation of treatments to patient and tumour characteristics (precision medicine). One pre-requisite for these approaches to be successful is to have tools for an accurate evaluation/follow-up of tumour load, physio-pathologic changes in the tumour, and/or spread in time and space of the disease course.

By providing a valuable alternative to gold-standard biopsies, non-invasive molecular imaging approaches including optical imaging, positron emission tomography (PET), single photon emission computed tomography (SPECT) associated with anatomical computed tomography (CT), or magnetic resonance imaging (MRI) imaging are particularly relevant approaches that could represent a step forward for the pre-selection of patients having the highest chance to benefit from the targeted therapy, for the follow-up of treated patients and of the disease evolution as well as for the detection of lesions not accessible for biopsies.

Nanobodies meet many criteria as non-invasive molecular imaging probes, notably, they have a high affinity and are small size compatible with fast targeting and optimal tissue penetration, and have a rapid blood clearance assuring a high tumour-to-background ratio in a short time frame (1). Their use in different molecular imaging modalities (optical, nuclear, ultrasound) has been rapidly expanding with a growing number of targets (1, 2). Some of them are currently in clinical trials for positron emission tomography imaging (PDL-1, HER2, and Macrophage Mannose Receptor (MMR)) (3).

Mesothelin (MSLN) is only expressed, at a low level, in healthy mesothelial tissues (pleura, peritoneum, pericardium), and is also highly expressed in several human cancers, notably in cancers characterised by aggressive phenotypes and poor prognoses such as mesothelioma, pancreatic, lung, ovarian cancers, acute myeloid leukaemia or triple negative breast cancers (4, 5). Although its physiological role is still poorly understood, a growing body of preclinical and clinical data demonstrates the active role of MSLN expression in the processes of malignant transformation, aggressiveness, and chemoresistance potentially through the Wnt/NF-κB/ERK1/2/Akt signalling pathways (6). These functions are mainly associated with its binding to MUC16, the only known ligand of MSLN. For these reasons, MSLN has been gaining momentum in recent years, leading to the emergence of a variety of targeted therapeutic approaches at various stages of development, including antibody-based therapies (ADC or immunotherapy), vaccine or cellular (CAR-T cells) approaches (6–8), mainly for malignant mesothelioma and ovarian cancer indications. Currently, diagnosis and treatment monitoring of MSLN-positive tumours rely mainly on blood tests for the presence of soluble mesothelin-related peptide (SMRP) and/or immunohistochemistry for tissue biopsy. However reports about the reliability and/or sensibility of SMRP tests are contradictory, at least for some types of cancers (9) and biopsies are invasive procedures that cannot be repeated on a regular basis. Non-invasive imaging of MSLN-positive tumours could expand the arsenal of tools for diagnosis, stratifying patients as potential responders to MSLN-targeted therapies or monitoring the response to treatment and/or the disease evolution or for fluorescence-guided surgery for instance.

In this respect, MSLN imaging has been reported for different types of cancers mainly in preclinical models (10) and in 2 clinical studies (11, 12). Most of these studies involve conjugated monoclonal antibodies, which have the disadvantage of requiring a long lag time (24-96 h) before obtaining a satisfactory tumour/background contrast, limiting their routine use.

In this study, we generated a new high affinity nanobody (S1) targeting MSLN whose binding is not impeded by the presence of MUC16, to be used for either non-invasive optical or PET imaging. We demonstrated that ATTO-647N- and ^68^Ga-conjugated S1 can specifically target MSLN-positive tumours *in vivo*, highlighting its potential for non-invasive imaging of MSLN as a companion test for MSLN-targeting therapies.

## Materials and methods

### Cells lines

Cell lines were obtained from the American Type Culture Collection (Manassas, VA) and submitted to no more than 20 passages, which were routinely tested for mycoplasmas (MycoAlert Mycoplasma Detection Kit, Lonza) and cultured in a humidified environment at 37°C and 5% CO_2_. Ovarian cancer cell lines OVCAR 3 (ATCC^®^ HTB-161™) were cultured in RPMI 1640 supplemented with 20% foetal bovine serum (FBS) respectively and 0.1% bovine insulin. A1847 (Cellosaurus, CVCL_9724) cells and AsPc1 (ATCC^®^ CRL-1682™) were cultured in RPMI 1640 supplemented with 10% FBS. HEK 293T HEK 293T/17 (ATCC^®^ CRL-11268™), MDA-MB-231 (ATCC^®^ HTB-26™), and HeLa (ATCC^®^ CRM-CCL-2™) were maintained in Dulbecco modified Eagle medium supplemented with 10% FBS.

### Cell transfection for MSLN expression

Adherent HEK293-T cells (70-80% confluence) were transfected with GFP-MSLN (Human Mesothelin/MSLN Gene ORF cDNA clone expression plasmid C-GFPSpark tag, SinoBiologicals) using Lipofectamine™ 3000 Transfection Reagent (Invitrogen) diluted in Opti-MEM (Gibco) according to the manufacturer’s instructions. MSLN expression was evaluated 12-24h hours post transfection by flow cytometry using 10 nM mAb K1 (Genetex) and Alexa647-conjugated goat anti-mouse (1/300, Miltenyi).

### Antibody penetration in tumour spheroids

A1847-derived tumour spheroids were generated as previously described (13). Briefly, cells were harvested and seeded (10^4^ cells/well, > 95% viability) into Corning^®^ Costar^®^ 96 well ultra-low attachment round bottom plates. Plates were centrifuged at 400xg for 2 min and allowed to incubate at 37°C under standard conditions. After 3 days, ATTO-647N-conjugated nanobodies (50 nM) were added to the wells. Spheroids were carefully washed with PBS 1x at the indicated time and then fixed with 4% paraformaldehyde for 30 min at room temperature. Spheroids were first washed with PBS 1x/2% BSA and then PBS 1x before clearing with CUBIC I solution (25% urea, 25% N,N,N’,N’-tetrakis (2-hydroxypropyl)ethylenediamine, and 15%Triton X-100) (14). Spheroids were embedded in warm 1% Low Melting Point agarose into thin glass capillaries and imaging was performed using a Zeiss LightSheet Z.1 Microscope and a 5x/0.16 objective. Data analysis was performed with the Imaris viewer software.

### Generation of anti-MSLN nanobodies by phage display

A nanobody library was constructed in E. coli TG1 strain after immunisation of a llama (Lama gluma) with the recombinant human MSLN (rhMSLN-His, R&D Biotechnology) as previously described (15, 16). The first round of selection was performed on rhMSLN (10 µg/ml) immobilised on Maxisorp 96-well plates. Before the second round of selection performed at 4°C on OVCAR-3 cells (22x10^6^ cells), non-relevant epitopes were masked with anti-HEK nanobodies as previously described (17). Individual TG1 colonies (186 clones) from the selection outputs (round 2) were randomly picked and grown overnight at 37°C in 2YTAG (2YT complemented with 100µg/ml ampicilline and 2% glucose) in 96-microwell plates for the screening step. Overnight cultures were used to inoculate fresh 2YTA medium. After growing for 2h at 37°C, the production of nanobodies was induced by the addition of 0.1 mM IPTG and overnight growth at 30°C. Supernatants were harvested and used for screening on GFP-hMSLN-transfected HEK 393 T cells using non-transfected HEK 293 T cells as a negative control. The binding of nanobodies was detected using an anti-HIS antibody (Novagen, 1/500) and Alexa 647-conjugated goat anti-mouse IgG (Alexa 647-GAM, 1/300 Miltenyi). Selected clones were sequenced to identify distinct nanobodies (Genecust).

### Production and purification of Nb S1

After the transformation of E. coli BL21 DE3 strain by positive phagemids, the nanobody production was performed as described in Behar et al. (16) after induction by 0,1 mM IPTG. Bacteria were pelleted and lysed in Bugbuster lysis buffer (Merck Milliopore) supplemented with benzonase (25 U/ml, and lysozyme (20 µg/ml). The his-tagged nanobodies were purified by affinity chromatography on TALON superflow™ cobalt resin (GE Healthcare, 28-9575-02) followed by a desalted step on Sephadex G-25 resine (Cytiva, 17085101). Nanobodies were stored in PBS. The protein concentration was determined spectrophotometrically (Direct Detect^®^). Protein purity was evaluated by SDS-PAGE on a 4-20% Mini-PROTEAN^®^ TGX Stain-Free™ Protein stain free gel (BioRad) under reducing conditions. Western blotting was performed on nitrocellulose membrane using a trans-Blot Turbo Transfer System (BioRad). Precision Plus Protein™ unstained and Prestained Standards (BioRad) were used for SDS-PAGE and Western blot respectively.

### Sortase A mediated conjugation

To generate the sortag-nanobody, the coding sequence of the sortase A recognition sequence LPETG was introduced upstream of the C-terminal His-tag in the nanobody coding sequences. The resulting sequences were cloned in frame behind the pelB leader sequence in the pJF55 vector. Plasmids were transformed in E. coli BL21DE3 for standard protein expression and nanobody-sortag were purified by size exclusion chromatography (Superdex™ Increase 75 10/300GL (GE Healthcare)). The integrity and binding capacity of nanobody-sortag were verified by SDS-PAGE 4-20%, flow cytometry on A1847 cells, and biolayer interferometry.

Pentamutant sortase A plasmid (Addgene, plasmide #75144) was modified to replace the 6HIS tag by the Twin-Strep-tag (SA-WSHPQFEK-(GGGS)2-GGSA-WSHPQFEK) and the resulting enzyme was produced in E. coli BL21DE3 and purified by affinity chromatography on Strep-Tactin XTSuperflow^™^ resin (IBA lifescience^®^) according to manufacturer’s instructions. The peptides for sortase A-mediated ligation, GGGWWSSK(NODAGA)-OH, and H-GGGYK-biotin were purchased from Pepscan. NODAGA: 2,2′-(7-(1-carboxy-4-((2,5-dioxopyrrolidin-1-yl)oxy)- 4-oxobutyl)-1,4,7-triazanonane-1,4-diyl)diacetic acid). The sortase reaction was performed at 25°C for 2h in 50 mM Tris-HCl, 150 mM NaCl, and 10 mM CaCl_2_ buffer pH 7,5 using a molar ratio of sortase/Nb-sortag/peptide-NODAGA of 1/10/100. The sortase was depleted on Strep-Tactin XTSuperflow™ resin and unbound peptide-NODAGA was removed by size exclusion chromatography on Superdex™ Increase 75 10/300GL (GE Healthcare) with PBS 1x pH 7.5 as running buffer.

The integrity and binding capacity of nanobody-sortag-Biot/NODAGA were verified by SDS-PAGE 4-20% and flow cytometry on A1847 cells, respectively. Matrix-Assisted Laser Desorption Ionization Time-of-Flight Mass Spectrometry (MALDI-TOF MS) was carried out for assessing the presence of biotin or NODAGA groups.

### Flow cytometry experiments

All flow cytometry experiments were performed on a MACSQuant cytometer (Miltenyi Biotec) using V-bottom 96-well microtiter plates. Cells were gated on single-cell populations and 10^4^ events were collected for each sample. Data were analyzed with the MACSQuant software and the results were expressed as the median of fluorescence intensity.

### Binding affinity measurements on cells

MSLN-positive cells (2x10^5^ cells/well) were first saturated using PBS/BSA 2% for 1 h at 4°C to avoid non-specific binding and incubated with serial dilutions of anti-MSLN nanobody for 1 h at 4°C in PBS/BSA 1%. Bound nanobodies were detected by staining 1 h at 4°C with a mouse anti-HIS mAb (1/500, Novagen) and Alexa 647-GAM. Three washes in PBS/BSA 2% were performed between each incubation step. The binding of monoclonal antibody K1 (Genetex) was detected with an Alexa-647- GAM. An irrelevant nanobody and/or Alexa 647 GAM were used as negative controls.

### Binding affinity measurements on recombinant antigen

rhMSLN-HA-His (5 µg/ml) was coated on Nunc MaxiSorp™ ELISA 96 flat bottom microplates overnight at 4°C in PBS. After a saturation step with PBS/5% milk for 1 hour at RT, serial dilutions of nanobodies were added for 1 h at 4°C under shaking. Bound nanobodies were detected using an anti-cmyc antibody followed by an HRP-conjugated goat anti-mouse IgG (1/1000). The detection of peroxidase activity was performed using TMB (3,3’,5,5’-Tétraméthylbenzidine - KPL) substrate, and OD_450nm_ was measured on a Tecan Infinite^®^ M1000 plate reader after the addition of HCL 1N stop solution.

In both cases, the curves were fit with a sigmoidal dose–response equation, and EC50 values were calculated using the Prism 5 software.

### Biolayer interferometry

Bio-layer interferometry (BLI) on the Octet R2 system (Pall ForteBio) was used to measure binding kinetics between nanobody and biotinylated rhMSLN-Fc. Streptavidin biosensor was rehydrated in binding buffer (PBS supplemented with 1% BSA and 0.05% Tween 20) for 10 min at 25°C. Biotinylated rhMSLN (10µg/ml) in binding buffer was bound to streptavidin sensor for 120 s. After an equilibration step in binding buffer for 30 s at 25°C, the MSLN-bound sensor was exposed to various concentrations of nanobody (50, 12.5, and 3.13 nM) for 300 s (association step) and then to a nanobody-free binding buffer for 300 s for the dissociation step. Kinetic constants were determined by fitting data with a 1:1 stoichiometry using the Octet analysis studio software.

### Expression of MSLN and MUC16

MSLN and MUC16 binding capacity of tumour cell lines was quantified by DAKO QIFIKIT (DAKO Cytomation), according to the manufacturer’s protocol. Briefly, tumour cells were first labelled with anti-MSLN mAb K1 (150 nM, Genetex) or anti-CA125 mAb X75 (100 nM, ThermoFisher Scientific) on ice for 60 min. After several washes in PBS-BSA 2%, FITC-conjugated goat anti-mouse F(ab’)2 antibody diluted 1:50 (QIFIKIT, Agilent Dako) was used for labelling of both calibration and set-up beads (QIFIKIT, Agilent Dako) as well as tumour cells. Set-up beads were used to establish the window of analysis and the calibration beads were used to construct a calibration curve. The MSLN or MUC16 densities were determined by extrapolation on the calibration curve and expressed as specific antibody-binding capacity units after subtracting the background from the isotype control.

### Epitope mapping

Epitope mapping was carried out by ELISA using different recombinant MSLN home-made constructs corresponding to MSLN domain 1 (aa296-390, DIH-Fc), truncated domain 1 (aa 296-354, DIL-Fc), domain II/III (aa391-598, HA-His-tagged DII/DIII) based on the putative MSLN structure (18). All the constructs were produced in the eukaryotic system using the Gibco™ EXPI 293™ Expression System Kit (Fisher Scientific) following the procedure provided by the manufacturer and purified by affinity chromatography on a GE Talon^®^ Superflow™ cobalt resin column. Correct conformation of the DII/DIII protein was assessed using the SD1-Fc fusion protein described by Tang et al. (19). ELISA procedure was as described above and the concentration of nanobodies was fixed at 100 nM. Affinity measurements were performed on D1H-Fc and D1L-Fc by ELISA and data were analyzed with GraphPad Prism software.

### Epitope binning

MSLN nanobody were site-specifically biotinylated using sortase A-mediated conjugation (eSrtA, Addgene) and a GGGYK-biotin peptide (Pepscan) at a molar ratio of nanobody-sortag/sortase/peptide-biotin of 1/0.1/20. For competition experiments A1847 cells (2x105 cells/well) in PBS/BSA 1% were incubated for 1 h at 4°C with serial dilutions of MSLN nanobody and their biotinylated counterpart at their EC50. After 2 washes in PBS/BSA 1%, cells were incubated with streptavidin-Alexa FluorX® (1/300, BioLegend) and analysed by flow cytometry. Data were analysed with GraphPad Prism software. Epitope binning was also analyzed by biolayer interferometry using an Octet R2 system (Sartorius). Biotinylated human MSLN (10 µg/ml) was immobilised on streptavidin sensors. In the first step, antibody 1 (Nbs A1 or S1 or amatuximab) in PBS (100 nM) was bound for 500 seconds to MSLN-bound biosensors. In the second step, antibody 1 (100 nM) was mixed with antibody 2 (100 nM, Nbs A1, S1, or amatuximab) to avoid a potential displacement of the already bound antibody 1. No wavelength shift should be observed if both antibodies share the same epitope.

### MSLN/MUC16 blocking assays

To test the blocking property of MSLN nanobodies, hrMSLN-Fc was mixed with a 10-fold molar excess of anti-MSLN- or irrelevant nanobodies in PBS/BSA 1%. After a 30 min incubation at room temperature, the mixture was added to Nunc maxisorp 96-well plates pre-coated with rhCA125 (5µg/ml, R&D Systems^®^) for 1 h at RT. After 3 washes in PBS/Tween 0.1% followed by 3 washes in PBS, MSLN-Fc binding was detected by the addition of HRP-conjugated goat anti-human Ig (1/1000, Life Technologies) for 30 min at RT. The detection of peroxidase activity was performed as described above. The percentage of binding inhibition was determined using the following formula: % blocking=100*(1-(A450nm assay/A450nm”no Ab condition”). (n=3)

### Heterotypic cancer cell adhesion assay

OVCAR 3 cells (4 × 10^4^) were seeded in triplicate in black Corning^®^ 96 well flat clear bottom black microplates (3603). Two days later, GFP-MSLN transfected HEK 293 T cells (3 × 10^5^) were incubated in the presence or absence of anti-MSLN/ANef nanobodies (1 µM) at 4°C, 30 min in RPMI 10% FCS then added to the OVCAR-3 monolayer for 1 hr at 37°C. GFP signals were recorded at 508 nm before and after 7 washes in PBS using a fluorescent plate reader (Tecan Infinite^®^ M1000 - Life Technologies). The percentage of adhesion was calculated using the formula: (FAW/FBWsample)/(FAW/FBWmedium) *100 as described by Bergan et al. (20) with FAW= fluorescence after washes and FBW=fluorescence before washes. Incubation without antibody corresponds to the reference condition (n=3).

### Microbial transglutaminase mediated ATTO 647N labelling

ATTO 647N labelling was performed using the Zedira TGase Protein Q-Labelling kit (L107) according to the manufacturer’s protocol. A size exclusion chromatography on GPC column (L107 kit) was carried out to remove the excess of ATTO 647N and the degree of labelling (DOL, dye-to-protein ratio) was calculated as follows: DOL= (A646nm*Eprot)/((A280nm-A646nm*CF280)*Emax) with A646nm: Absorbance at 646 nm, A280nm: Absorbance at 280nm, Eprot: Extinction coefficient of the protein in M-1cm-1, CF 280: Attenuation coefficient of ATTO647 at 280 nm (= 0.03) and Emax: Extinction coefficient of the fluorophore. The integrity and functionality of ATTO 647N conjugated nanobodies were assessed by 4-20% SDS-PAGE and flow cytometry.

### Internalisation assay

A1847 cells (1.5x10^4^ cells/well) were grown on a glass coverslip immerged in 24 well plates for 2 days at 37°C. After washing the cells with PBS, a saturation step was carried on in PBS/BSA 3% for 1 h at 4°C. Then the cells were incubated for 1h at 4°C or 37°C with HA-His-tagged nanobodies (500 nM). The coverslips were washed with PBS-BSA1%, fixed with 4% p-formaldehyde for 30 min at RT, and permeabilised in PBS/0.5% Triton-X100 for 10 min before a 1 h-incubation with AlexaFluor 488-conjugated anti-HA antibody (1/200, LifeTechnologies) at RT. After several washes, the nuclei were stained with DAPI (1/2000 ThermoFischer) for 5 min. Fluorescence was evaluated using an Apotome fluorescent microscope (Zeiss), magnification: x63.

### Animal experiments

In accordance with the European Directive 2010/63/EU on the use of animals for scientific purposes, all procedures using animals were approved by the Institution’s Animal Care and Use Committee of Aix-Marseille University. The corresponding Project Authorizations (agreements APAFIS#28902 (TrGET platform) and #32157 (CERIMED)) were delivered by the French Ministry of Research and Higher Education. The animals were housed in enriched cages placed in a temperature-and hygrometry-controlled room with daily monitoring. Food and water were provided ad libitum.

### *In vivo* fluorescence Imaging

A1847 MSLN^pos^ (5x10^6^ cells) and MDA-MB-231 MSLN^low^ (4.4x10^6^ cells) cells in a 1/2 (v/v) Matrigel (Corning Life Sciences, Bedford, MA, USA) suspension were implanted subcutaneously in 8-week-old female NOD-SCID IL-2Rgamma(null) (NSG) mice (n=24/cell line, n=8/group) and grown until all the tumours reached an average volume between 250 and 300 mm^3^. ATTO647N™ conjugated nanobodies (27 µg with an average DOL of 0.57) were injected *via* the tail vein and *in vivo* whole body fluorescence images were acquired using a Photon Imager (BioSpace Lab), at the following time points: 1, 6, and 24 h. Background fluorescence was determined on a xenografted mouse without antibody. Fluorescence signals within the regions of interest are expressed as photons per square centimetre per second per steradian (ph/cm^2^/s/sr) and determined using the following formula: Signal from ROI tumour - signal from ROI negative. After the final timepoint, animals were euthanised by cervical dislocation, and fluorescence imaging of individual organs was performed. Results were expressed as ph/cm2/s/sr (photon per square centimetre per second per steradian) or as a percentage of total signal (100*(organ signal - signal of non-injected mouse)/total fluorescence). The optical fluorescence measurements were performed with the PhotoAcquisition software (Biospace Lab), on 2 or 3 mice belonging to the same experimental group at the same time. We used identical acquisition parameters for all animals and for all time points (1, 6, and 24h). The analyses were carried out using the M3Vision software (Biospace Lab). The analysis parameters used for the 1 h time point were different from those used for the 6 and 24h time points. However, for each time point, the analytical parameters are the same for all animals. The fluorescence scales are indicated in the figures.

### MicroPET/CT imaging

A1847 MSLN*^pos^* (10x10^5^ cells) and MDA-MB-231 MSLN^low^ (5x10^5^ cells) cells in a 1/1 (v/v) Matrigel (Corning Life Sciences, Bedford, MA, USA) suspension were implanted subcutaneously in 6-week-old female NMRI-Foxn1<nu>mice (A1847, n=20; MDA-MB-231, n=20) and grown until the tumours reached an average volume of 100 – 300 mm^3^ (average sizes of MDA-MB-231 and A1847 tumours were 197 +/-147 and 266 +/-186 mm^3^ respectively). For radiolabeling, nanobody S1 (50 µg in PBS) was first diluted in fresh 4M NH4OAc pH 5,0 (50 µl) and then mixed with 500 µl of Gallium-68 chloride ([^68^Ga]GaCl). The mixture was stirred for 10 min at room temperature. The radiochemical purity was assessed by radio-thin-layer chromatography (solid phase: ITLC-SG) in two different mobile phases (sodium citrate 1 M, pH 5,0 and a solution of methanol/1M NH4OAc (1:1, v:v)) using a miniGITA radio-TLC scanner detector (Raytest, Straubenhardt, Germany). Radiolabeling stability was evaluated by iTLC after incubation of ^68^Ga-nanobody in human plasma or NaCl 0,9% at 37°C for 30 and 120 min after radiosynthesis.

For dynamic microPET/CT imaging, mice were maintained under 1.5% isoflurane anaesthesia and imaged for 2 hr, immediately after intravenous injection of 68Ga-nanobody S1 (5-7 MBq/mouse) in the tail vein. A catheter 26G was placed into the tail vein of the mice to facilitate a rapid radiotracer injection. For the static experiment, microPET images were acquired for 20 min, 2h after intravenous injection of the radiotracer (5 MBq/mice). Image acquisition was performed on a NanoScan PET/CT camera (Mediso, Budapest, Hungary). After each PET recording, micro-CT scans were acquired for anatomical coregistration. Region-of-interest (ROI) analysis of the PET signal was performed on attenuation- and decay-corrected PET images using VivoQuant v.4.0 software (InVicron, Boston, USA) and tissue uptake values were expressed as a mean percentage of the injected dose per gram of tissue (%ID/g) ± SD. At the end of the static experiments, mice were sacrificed by cervical dislocation, and the radioactivity of individual organs was measured using a gamma counter (Wizard™ from Perkin Elmer). For the blocking experiment, mice (n=6) were pretreated with a 150-fold molar excess of unlabelled S1 (i.v.). Results were expressed as the percentage of the injected dose per gram of tissue (%ID/g) +/- SD.

### Statistical analysis

Statistical analyses were performed with GraphPad Prism software (V5.01). Unpaired two-tailed t-tests or Mann-Whitney tests were used to do a pair-wise comparison. Two-way ANOVA followed by Turkey *post hoc* test were used for *in vivo* competing experiments. All statistical tests and resulting *P* values are indicated in the figure panels or the figure legends. *P*-values below 0.05 were considered statistically significant.

## Results

### Generation of Nb S1

MSLN nanobodies were isolated from a phage-nanobody library generated after immunisation of llama with the mature recombinant human MSLN protein. Two successive rounds of selection were performed, first on recombinant MSLN protein and then on high-grade serous ovarian adenocarcinoma cell line OVCAR3. After screening for MSLN binding on HEK 293T cells transfected with human mature MSLN, three clones displaying different sequences (A1, C6, S1) were isolated, two of which (A1 et C6) have been described previously (15). The clone S1 was therefore selected for further characterisation. As Nb A1, Nb S1 displayed the hallmark residues of the VHH genes in the framework 2 regions (21). Nb S1 was produced at a large scale in E. coli as previously described (16) and purified by affinity chromatography on TaLon and size exclusion.

### Nb S1 binding capacity and specificity

**Figure 1:**
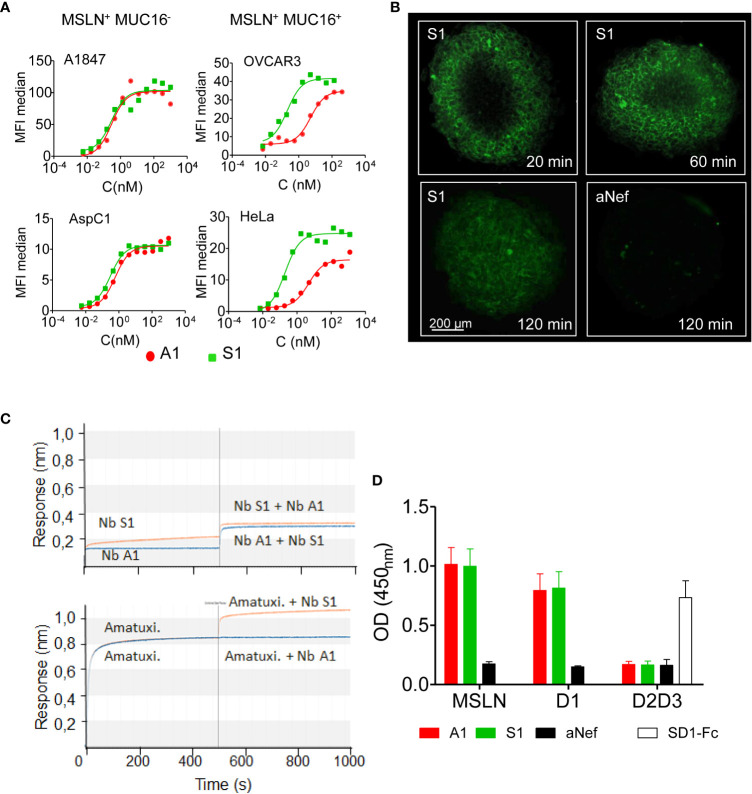
Binding properties of Nb S1. **(A)** Binding of Nb S1 and A1 on A1847, OVCAR3, AspC1 and HeLa cells measured by flow cytometry. Binding was detected with a mouse anti-HIS mAb followed by an Alexa647-conjugated goat anti- mouse IgG. Curves were analyzed using the one site total binding (PRISM Graphpad). **(B)** Representative images of A1847 spheroids cross-sections illustrating infiltration of ATTO-647N-labeled S1 or anti-Nef nanobodies (100 nM, green fluorescence). Images were analyzed using Imaris viewer software. **(C)** Epitope binning was performed by biolayer interferometry using biotinylated MSLN immobilised on streptavidin sensors. Antibodies were allowed to bind in sequential steps. Upper panel, epitope binning between A1 and S1 nanobodies. First step: incubation of MSLN-coated sensor with 100 nM nanobody A1 (blue line) or S1 (orange line) for 500 sec. Second step: incubation with a mix of Nb A1 and S1, 100 nM each. Lower panel, epitope binning between Nb S1 and amatuximab. Same protocol as above. **(D)** Epitope mapping on recombinant MSLN and isolated domains by ELISA. Domain 1-Fc fusion (D1 aa: 296-390), HA/HIS-tagged domains 2 and 3 (D2D3: aa391-598). Binding of Nb (100 nM) on immobilised mesothelin derivatives was detected using anti-cmyc Ab and HRP-conjugated goat anti-mouse IgG (n=9). SD1-Fc antibody was used as quality control of recombinant D2D3 fusion protein (19).

The capacity of nanobody S1 to target MSLN+ cells was evaluated by flow cytometry on a panel of cancer cell lines from different cancers, expressing various levels of MSLN and of its ligand MUC16 (**Figure 1A**; **Supplementary Figures 1A, B**). The binding of Nb S1 was efficient on all cell lines as in all cases more than 90% of cells were labelled. The titration curves demonstrated that Nb S1 binds mature MSLN in a dose-dependent manner with an apparent affinity in the nanomolar range regardless of the presence of MUC16 (**Table 1**). The kinetic parameters of Nb S1 (**Table 2**) were determined by bio-layer interferometry. As shown in **Figure 1B**, a rapid distribution of the of ATTO 647N-labeled Nb S1 inside A1847-derived spheroids was observed compared to irrelevant aNef Nb, leading to a homogenous labelling throughout the entire spheroid in 2 hrs. The specificity of binding was confirmed by the absence of binding in the presence of an excess of soluble MSLN ectodomain. As well, ELISA on murine and human recombinant MSLN demonstrated that, unlike Nb A1, no binding of Nb S1 was observed on the murine protein.

**Table 1:** Binding parameters on MSLN-positive cell lines.

	EC_50_ (nM)		Specific Binding Capacity*	
	Nb A1	Nb S1	MSLN	MUC16
A1847	0.79+/-0.57	0.35+/-0.12	103251	3100
AsPc1	0.42+/-0.22	0.26+/-0.08	30665	3692
**Ovcar3**	5.42+/-3.76	0.30+/-0.27	50714	295003
**HeLa**	4.48+/-0.48	0.17+/-0.06	130327	553929

*Qifikit data. In bold, cell lines expressing also MUC16.

**Table 2:** Kinetic parameters of anti-MSLN Nbs and derivatives.

	K_D_ (M)	k_on_ (M^-1^s^-1^)	k_off_ (s^-1^)
S1-myc-HIS	1.468x10^-9^	6.208x10^5^	9.114x10^-4^
A1-myc-HIS	0.8581x10^-9^	16.43x10^5^	14.10x10^-4^
S1-LPET-HIS	1.30x10^-9^	2.98x10^5^	3.9x10^-4^
S1-sortagNODAGA	1.85x10^-9^	6.10x10^5^	1.13x10^-3^
S1-ATTO647N	7.63x10^-9^	1.55x10^5^	1.18x10^-3^

### Epitope characterisation

The epitope targeted by Nb S1 was first investigated by bio-layer interferometry using streptavidin sensors pre-coated with biotinylated human mesothelin. The sensorgrams (**Figure 1C**) showed that Nb S1 binds to an epitope distinct from that of Nb A1 or amatuximab and in the same way whatever the order of its addition in the reaction. As expected from previous data on Nb A1 (13, 15), Nb A1 and amatuximab recognise the same epitope which overlaps the MUC16 binding site. Competition experiments on A1847 cells using biotinylated and non-biotinylated A1 and S1 confirmed these results as no competition was observed between Nb S1 and biotinylated Nb A1 or vice versa.

The immunoblotting experiment revealed that Nb S1 was able to detect human recombinant MSLN on western blotting in reducing conditions suggesting that Nb S1 recognises a linear epitope, as Nb A1 and mAb K1.

Next, truncated mutants of mesothelin were constructed based on the hypothesis that mature MSLN is organised in 3 distinct domains (**Supplementary Figure 1C**): a membrane distal domain I (residues 296–390), domain II (residues 391–486) and a proximal membrane domain III (residues 487-581) (22). Domain 1 (residues 296–390, D1) and truncated domain 1 (residues 296–354, D1L) were generated as Fc-fusions while the D2/D3 fusion protein was generated as a monomeric HA-HIS-tagged protein. The binding of Nb S1 was assessed by ELISA on immobilised mature and truncated MSLN (**Figure 1D**). Nb S1 binds both mature rhMSLN and D1 but not D2-D3 indicating that Nb A1 and Nb S1 bind the membrane distal domain. The apparent affinity of Nb S1 was assayed by ELISA on mature MSLN, D1-Fc, and D1L-Fc. While the apparent K_D_ of Nb A1 on the 3 targets were similar (**Table 3**), Nb S1 displayed a significant decrease of apparent affinity for D1L-Fc, highlighting the importance of amino acids 359-390 for Nb S1 binding to MSLN.

**Table 3:** Apparent affinity on immobilised full size MSLN and MSLN domains 1.

K_D_ (nM)	Nb A1	Nb S1
rhMSLN	2.1+/-0.3	1.7+/-0.3
D1	9.3+/-1.9	3.6+/-0.6
D1L	3.4+/-0.9	55.1+/-27.4

### Binding of Nb S1 is not altered by MUC16/MSLN interaction

**Figure 2:**
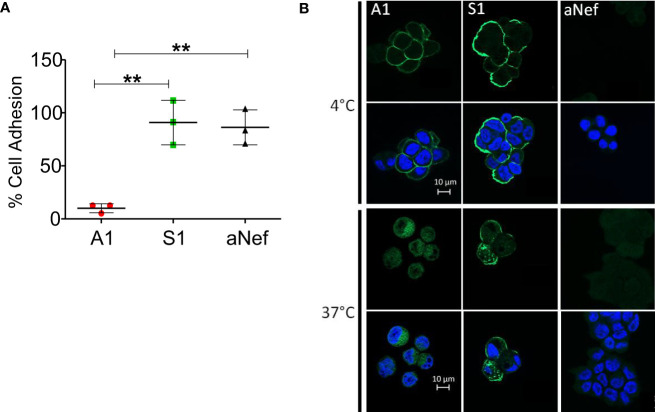
S1 Nb properties. **(A)** Heterotypic cell adhesion assay between OVCAR-3 and GFP-MSLN transfected HEK 293 T. The protocol is sketched in the upper panel. Cell adhesion was measured in the presence or not of 1μM Nb A1, S1 or irrelevant aNef. The percentage of adhesion is calculated relative to the control condition without antibody (n=3). Values correspond to means +/- standard deviation of 3 independent experiments. The p-values were calculated with two-tailed unpaired t-test, ** p-value < 0.01. **(B)** Nanobody internalisation was observed by ApoTome fluorescence microscopy. A1847 cells were incubated with 500 nM Nbs at 4°C or 37°C. The nucleus was stained using DAPI. The scale bar equals 10µm. Objective 63x.

Mesothelin is used both as a tissue marker and as a serum marker in association with CA-125 in several cancers. Many antibodies targeting MSLN recognise an epitope located in the MUC16/MSLN binding site (22), suggesting that the detection of MSLN can be affected by the presence of MUC16. To determine whether MUC16/MSLN interaction is hindered by Nb S1 binding, we evaluated the adhesion of GFP-MSLN transfected HEK 293 T on OVCAR-3 (MSLN+, MUC16+) monolayer in the presence or absence of Nb S1. As shown in **Figure 2A**, the presence of S1 did not impede the adhesion between the 2 cell lines in contrast to A1 which blocked more than 90% of MSLN-transfected cells adhesion. Similar results were obtained by ELISA on plate-bound recombinant human CA125.

### Internalisation of anti-MSLN Nbs

To test whether Nb S1 is internalised upon MSLN binding, HA-His-tagged Nb S1 was incubated with OVCAR-3 cells at 37°C, a permissive temperature for internalisation, and at 4°C as control. As shown in **Figure 2B**, at 4°C, fluorescent staining was localised at the plasma membrane with both Nb A1 and Nb S1. However, A1 staining is fainter than with S1, which could be related to the expression of MUC16 on these cells. At 37°C, fluorescence signals were mainly localised in the cytoplasm, in favour of the internalisation of Nb S1 and Nb A1 upon MSLN binding.

### *In-vivo* fluorescence imaging

**Figure 3:**
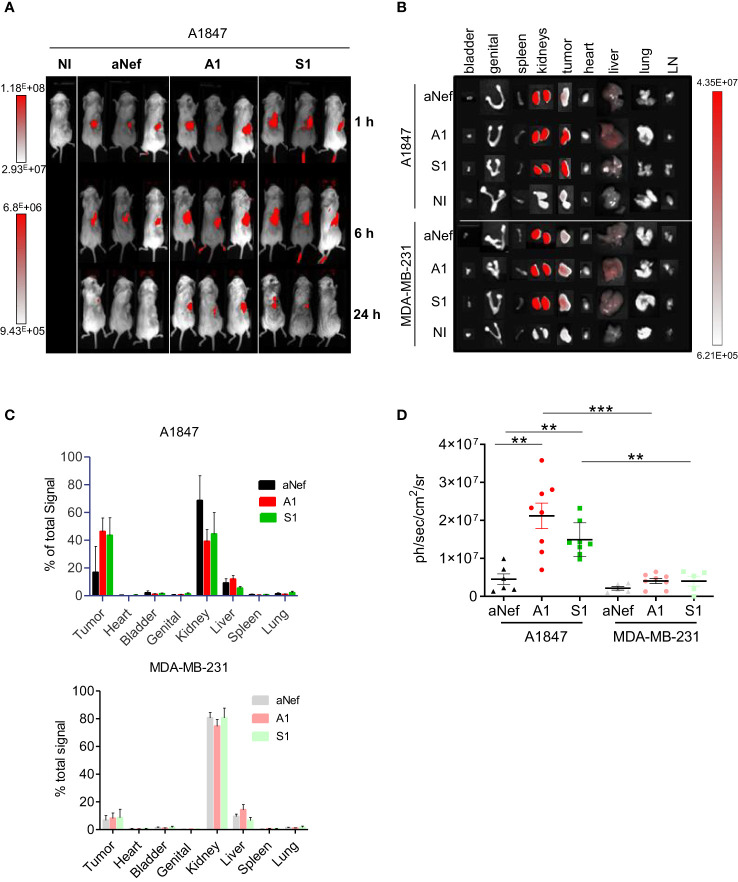
*In vivo* fluorescence imaging of MSLN-positive tumours. A1847 or MDA MB 231-xenografted mice were injected with 27 μg of ATTO 647N-S1 (n=6), -A1 (n=8), or irrelevant aNef (n=8) nanobody. **(A)** Representative whole body fluorescence imaging 1, 6 and 24 h post i.v. injection of ATTO 647N-conjugated nanobodies A1, S1 or irrelevant aNef in A1847. **(B)** Representative images of *ex vivo* fluorescence in resected tumours and major organs 24h post i.v. injection of ATTO 647N-conjugated nanobodies (A1, S1 or irrelevant aNef) in A1847 (upper panel) or MDA MB 231 (lower panel)-xenografted NSG mice. NI: non injected mouse, LN: lymph nodes. **(C)** *Ex vivo* quantification of tumour fluorescence of major organs and tumours 24 h post-injection. % of total signal = 100* (Organ signal of injected mice – organ signal of non-injected mice)/total signal. Error bars represent SD. **(D)** Ex vivo fluorescence measurements in resected tumours. Data are expressed as ph/sec/cm^2^/sr. Data were analysed using a two-tailed Mann-Whitney test using Graphpad Prism software: ** p <0,01. n=5-8, ***p-value <0.001 mice/group.

The targeting capacities of Nb S1 were evaluated *in vivo* by fluorescence optical imaging using ATTO 647N-conjugated MSLN nanobodies and NSG mice xenografted with either A1847 cells (MSLN^high^) or MDA-MB-231 (MSLN^low^) cells. The absence of impact of site-directed conjugation of MSLN Nb with ATTO 647N was checked by ELISA (**Supplementary Figure 2A**). Once tumours reached around 200-300 mm^3^, mice were injected intravenously with 27 µg of ATTO 647N-S1, ATTO 647N-A1, or irrelevant aNef-ATTO 647N nanobodies. Whole-body fluorescence images were obtained at different time points (1, 6, and 24 h) (**Figure 3A**). Accumulation of ATTO 647N-S1 and ATTO 647N-A1 in the A1847 tumours were visible as early as 1 h post-injection and up to 24 h (**Supplementary Figure 2B**; **Figure 3A**) in contrast to irrelevant-ATTO 647N nanobody. Quantification of fluorescence intensity at the tumour site revealed that the tumour uptakes of ATTO 647N-S1 and ATTO 647N-A1 nanobodies were significantly higher than that of aNef-ATTO 647N nanobody at all time points. In MDA MB 321 tumour-bearing mice, the fluorescence quantification showed that the 3 nanobodies generated similar signals that decreased in the same way over time (**Supplementary Figure 2B**).

To determine the biodistribution profiles of ATTO 647N-labeled S1 and A1, mice were sacrificed 24h post-injection, and *ex vivo* analysis of the fluorescent signal in resected tumours and organs was performed. Compared to other organs, kidney uptake was high, reaching up to 75-80% of the total fluorescence (**Figures 3B, C**), a well-described phenomenon for nanobodies due to their rapid blood clearance and retention by the kidney (23). As shown in **Figure 3C**, more than 40% of the total fluorescence signal was found in the A1847 tumours 24h post-injection of ATTO 647N-A1 and ATTO 647N-S1 compared to a mean of 17% with ATTO 647N-ANef. The fluorescence signal was significantly higher with ATTO 647N-S1 and ATTO 647N-A1 than with the irrelevant Nb in A1847 tumour-bearing mice (**Figure 3D**). In MDA-MB-231 tumour-bearing mice, the signal detected in the tumour in the presence of anti-MSLN Nb was not significantly different from that detected with the irrelevant Nb, highlighting the specific tumour capture in relation to the expression level of mesothelin.

### Sortase A mediated NODAGA conjugation and ^68^Ga Radiolabelling

After engineering anti-MSLN nanobodies for inserting a sortase A-recognition motif (LPETG), C-terminal specific conjugation with NODAGA chelator was performed using a pentamutant sortase A-twin-strepTag. The reaction efficiency was estimated by western blot through the decrease of nanobody-sortag-His tag. The reaction mixture was purified by a two-step process on Strep-Tactin XTSuperflow™ to remove the sortase followed by a size exclusion chromatography. Matrix-assisted laser desorption ionisation mass spectrometry (MALFI-TOFTOF) confirmed the presence of a major species corresponding to the NODAGA-conjugated S1 (14 910 Da) (**Supplementary Figure 3A**). IMAC purification on the Talon metal affinity column to remove the non-conjugated nanobody was not possible because the NODAGA cage chelates Co2+ ions with a fairly good affinity hindering the subsequent radiolabeling. Taking into account all steps, the overall conversion yield of unconjugated to conjugated nanobody was ranging from 30 to 60%.

The binding properties of NODAGA-conjugated Nb S1 on immobilised recombinant MSLN were also assessed by biolayer interferometry (**Supplementary Figure 3B**; **Table 2**). Also, competitive binding assays by flow cytometry confirmed that the NODAGA-conjugated S1 retained its binding properties on MSLN^+^ cells (**Supplementary Figure 3C**).

After several optimisation steps, NODAGA-Nb S1 was successfully radiolabeled with gallium-68 as evidenced by the radiochemical purity (RCP) of 97% in 1M sodium citrate pH 5.0 and 90% in 1M NH_4_OAc/MetOH evaluated by thin layer radio-chromatography (**Supplementary Figure 3D**) and an apparent specific activity of 2-3 GBq/mg. In human plasma the ^68^Ga-labelled nanobody was stable overtime 2 h at 37°C, (RCP>95% in 1M sodium citrate pH 5.0) (**Supplementary Figure 3E**).

### Non-invasive immunoPET/CT imaging

**Figure 4:**
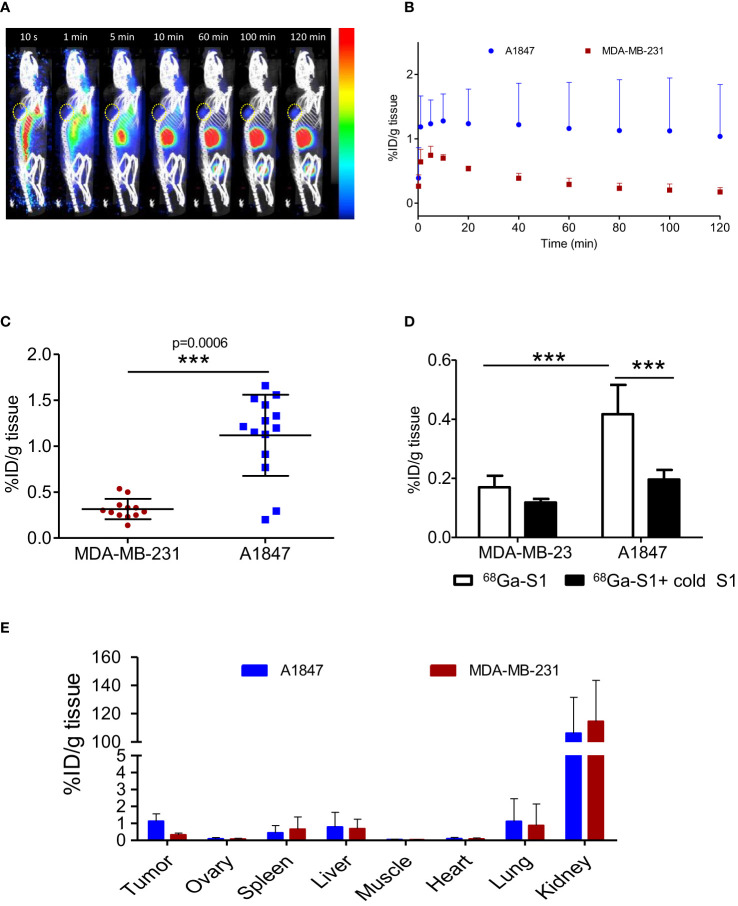
PET/CT imaging of MSLN-positive tumours. **(A)** Representative sagittal PET images of A1847 tumour-bearing mice after injection of [^68^Ga]Ga-NODAGA-S1 (5-7 MBq) during 2h dynamic scan. Yellow circle indicates the tumour. **(B)** Time activity curves (decay corrected) generated following radioactivity quantification from PET images (n=5 mice, A1847, n=3 mice, MDA-MB-231). **(C)** *Ex vivo* quantification of radioactivity in tumours. Data are expressed as a percentage of injected dose per gram of tissue after gamma-counting (n=14 mice for A1847 and 12 mice for MDA MB 231). Data were analysed using a two-tailed unpaired t-test: ***, p<0.001. **(D)** Competition experiment: tumour-bearing mice (n=6/group) were injected with [^68^Ga]Ga-NODAGA-S1 (5-7 MBq) and microPET images were acquired during 20 min, 2 h after intravenous injection of the radiotracer. The next day, the same mice were pretreated with a 150-fold molar excess of unlabeled S1 (1 mg, i.v.) 20 min before injection of [^68^Ga]Ga-NODAGA-S1 (5-7 MBq) (*n* = 6 mice/group) and images were acquired during 20 min, 2 h after intravenous injection of the radiotracer. Analysis of the PET signal was performed on attenuation- and decay-corrected PET images. Data were analysed using a two-way ANOVA followed by Turkey post-test: ****, p<0.0001. **(E)** *Ex vivo* biodistribution profile of [^68^Ga]Ga-NODAGA-S1 at 2 h post-injection in A1847 and MDA-MB 231-xenografted mice (n=14 mice for A1847 and 12 mice for MDA MB 231).

Mice bearing A1847 or MDA-MB-231 tumours were injected with [^68^Ga]Ga-NODAGA-S1 to determine its *in vivo* kinetics and distribution. As shown in **Figure 4A**, the nanobody was able to target MSLN+ tumours with a signal detectable 10 min post-injection, clearly visible 60 min post-injection and retained through 120 min scan. The time activity curves within the tumours presented in **Figure 4B** showed a rapid uptake in A1847 tumours that remained up to 2 h post-injection while MDA-MB-231 tumour uptake decreased over time. Kidneys and bladder showed high radioactivity accumulation in agreement with the well-described kidney retention and rapid blood clearance of nanobody.

PET static scans were also performed on mice bearing either A1847 or MDA-MB-231- tumours 2 h post-injection. *Ex vivo* biodistribution analyses demonstrated a significantly higher uptake of [^68^Ga]Ga-NODAGA-S1 in MSLN^high^ tumours (**Figure 4C**) compared to low expressing/negative MDA-MB-231 tumours. The specific tumour uptake was validated by a competition experiment with an excess of unlabeled Nb (**Figure 4D**). No significant difference was noted in the presence of a 150-fold molar excess of cold S1 in the MSLN^low^ tumours while a 50% decrease of the signal was observed in the MSLN^high^ tumours. The biodistribution of ^68^Ga-labeled nanobody was similar in all organs for the 2 groups of mice, except for the tumours (**Figure 4E**). As expected, high uptake of the radiotracer was observed in the kidneys in both MDA-MB-231 and A1847 tumour-bearing mice. Surprisingly, a higher uptake was observed in the liver and lung of 2-3 mice. One hypothesis to explain these results could be the presence of emerging tumour foci as in healthy mice no radiotracer uptake is observed in these organs.

## Discussion

In recent decades, a significant number of studies examined MSLN as a therapeutic target based on its differential expression profile between healthy tissue and tumours, and its prognostic impact. Concomitantly, non-invasive imaging tracers, mainly based on anti-MSLN antibodies, have also been under investigation (11, 24) as companion tools. Regarding MSLN imaging using nanobody-based radiotracer, very few preclinical studies are available in the literature on this topic (25–27), all of which are based on Nb A1 generated by our team (15). We have previously demonstrated the pertinence of anti-MSLN Nb (A1) as a versatile scaffold for generating diagnostic or therapeutic molecules (13, 15, 26). However, like most anti-MSLN mAbs, Nb A1 competes with MUC16 for MSLN interaction, which may decrease its targeting efficiency. The objective of this study was therefore to generate an anti-MSLN Nb whose binding properties are independent of the co-expression of the MUC16 ligand to improve the MSLN targeting and to evaluate its potential as a tracer for non-invasive PET/CT imaging.

We generated a new anti-MSLN Nb S1 that is able to bind all the tested MSLN-positive cell lines, with a high apparent affinity (EC50 = 0.35 +/- 0.12 nM) and regardless of the presence of MUC16. Analysis of the binding kinetics shows that Nb S1 dissociates more slowly than Nb A1, which could favour a higher residency time at the tumour site and its internalisation (28). Although targeting the membrane distal domain I of MSLN, Nb S1 does not compete with MUC16, nor with amatuximab. This property constitutes a major asset since it allows the monitoring of amatuximab-based therapies and current SS1-derived-drug conjugates without interference due to the presence of therapeutic reagents. Moreover, Nb S1 is efficiently internalised into the tumour cells upon binding to MSLN, a feature that can be exploited for radioimmunotherapy or for delivering chemotherapeutic molecules from a therapeutic perspective.

Fluorescence imaging is widely used in *in vitro* and preclinical settings for real-time visualisation of cell processes, and tissue structure or as a prerequisite to radiolabeling studies for imaging and/or vectorised internal radiotherapy. Most studies use near-infrared fluorescence dyes because of the low tissue absorption and low autofluorescence in this spectral range. Several studies have reported the use of anti-MSLN mAbs conjugated to infrared/near-infrared fluorochromes in different cancer pathologies (29). If the results are generally positive, in all these studies, a latency time varying from 24 to 96 h is necessary to obtain a satisfactory tumour/background ratio, because of the relatively long half-life of the mAbs (30). Up to now, only one preclinical study reports the use of anti-MSLN nanobodies for optical imaging (27). However, in this study coupling IRDye 680RD-labeled streptavidin to biotinylated Nb A1 failed to demonstrate the rapid clearance and high contrast imaging usually described with nanobodies, likely due to the biotin/IRDye 680RD streptavidin complex.

Taking advantage of reactive glutamine in the C-terminal c-myc tag of nanobodies, site-directed transglutaminase-mediated labelling of nanobodies was performed using the photostable red-emitting fluorescence dye, ATTO 647N. This strategy enables the controlled labelling of nanobodies in terms of fluorochrome payload (stoichiometric labelling) and localisation, two important parameters to maintain the targeting capacities of Nb. Whole-body fluorescence images showed that ATTO 647N-labeled MSLN Nbs but not the irrelevant Nb accumulated at the tumour site as early as 1h and up to at least 24 h in A1847^msln+^ -xenografted mice. *Ex vivo* fluorescence quantification in excised tumour and organs confirmed efficient and specific retention of ATTO 647N-labeled MSLN Nb (>40% of the total signal) in MSLN^high^ tumours up to 24 h. The similar tumour and organs biodistributions obtained with Nb S1 specific for human MSLN and Nb A1 that do cross-react with murine MSLN strongly suggest that the limited expression of MSLN on healthy tissues may not be a critical issue for clinical translation of Nb S1. This is supported by the fact that numerous MSLN-targeting therapeutic strategies currently investigated in clinical trials (antibodies, antibodies-derivatives, ADC, immunotoxin and CAR T cells) have been considered safe, without major off-target effects (for a review, 31).

A strong accumulation in the kidneys was observed as expected from the short half-life of Nb and the presence of 6His-tag. Different strategies can be considered to decrease renal reabsorption among which removing the his tag and/or injecting gelatin-based plasma expanders (Gelofusine) or positively charged amino acids (Lys, Arg) (32). These results confirm the potential of fluorescent-labelled MSLN Nb to detect MSLN tumours *in vivo* and the potential of same-day imaging, two features that can be exploited for fluorescence-guided surgery to help discriminate tumours from healthy tissue and precise excision of the tumour.

ImmunoPET/CT combines both the performance of PET/CT imaging (sensitivity, spatial resolution, morphological and functional data) and the exquisite antigen-binding properties of antibodies.

To achieve ^68^Ga-labeling of Nb S1 we used sortase-mediated conjugation of the NODAGA chelator, thus combining the removal of His-Tag and the site-directed C-terminal labelling, away from the paratope. The 3.7-fold higher uptake of [^68^Ga]Ga-NODAGA-S1 in MSLN^high^ A1847 than in MSLN^low^ MDA-MB-231 tumours associated with the competing effect of non-labelled S1 and a low off-target uptake confirmed the specific targeting of MSLN by Nb S1.

Despite the absence of His-tag, relatively important renal retention was still observed 2h post-injection. Improvements can be considered to increase tumour-to-background signals. Debie and colleagues (33) conducted an interesting study on the influence of the size and affinity (monovalent, bivalent, dimer) of nanobody-derived tracers on their ability to target and distribute homogeneously inside the tumour. They showed that these parameters can be adjusted according to the desired clinical applications, and notably that monovalency is a strong advantage for non-invasive imaging.

Further studies are required to evaluate the dose effect on tumour uptake and to investigate the targeting properties of anti-MSLN Nbs using orthotopic xenografts or different types of tumours as vascular permeability is a critical parameter. As well, optimising Nb S1 half-life in serum by fusion with an albumin binder of various affinities could also be an attractive alternative to achieve the best equilibrium between tumour accumulation and high contrast (34).

It is noteworthy that the use of two different mouse strains for imaging experiments allowed us to validate Nb S1 as an efficient non-invasive imaging agent in murine models regardless of their degree of immunodeficiency, which opens up the possibility, in the future, of evaluating immune cell engagers derived from these Nbs in the same preclinical model after human PBMC engraftment.

In conclusion, we developed a new Nb targeting MSLN, regardless of the presence of its ligand MUC16, and used it successfully to detect MSLN-positive tumours *in vivo*. We have shown, for the first time, the potential of an anti-MSLN nanobody for PET/CT imaging and demonstrated the selective accumulation of [^68^Ga]Ga-NODAGA-S1 in MSLN^+^ tumour, with high contrast images, shortly after systemic injection. These encouraging results open the way for the development of a matched/mixed theranostic approach as defined by Herrero-Alvarez et al. (35) by changing the diagnostic radioisotope by a therapeutic isotope such as ^177^Lu.

## Data availability statement

The original contributions presented in the study are included in the article/**Supplementary Material**. Further inquiries can be directed to the corresponding authors.

## Ethics statement

The animal study was reviewed and approved by Institution’s Animal Care and Use Committee (Aix-Marseille University). The corresponding Project Authorizations (agreements APAFIS#28902 (TrGET platform) and #32157 (CERIMED) were delivered by the French Ministry of Research and Higher Education.

## Author contributions

AbB and DM contributed equally to this manuscript and share the first position. Experiment design: AbB, DM, and BK. Phage display, *in vitro* Nb characterisation, Nb conjugation: AbB and DM with input from LD. Radiolabeling and PET imaging: LB, AhB, and BG with input from AbB. *in vivo* fluorescence imaging: AG and RC. Analysis and interpretation of data: AbB, DM, PC, AhB, BG, RC, and BK. Study conception and coordination, BK with input from PC. Supervision: BK and PC. Manuscript writing: BK and AbB with input from all authors. All authors contributed to the article and approved the submitted version.
