# Supplementary material for: Rapid nanobody-based imaging of mesothelin expressing malignancies compatible with blocking therapeutic antibodies
Source: Front Immunol. 2023 Jun 14;14:1200652. doi: 10.3389/fimmu.2023.1200652 (PMC10303918; doi:10.3389/fimmu.2023.1200652)
Supplement: Supplementary file 1 [file DataSheet_1.docx]

Supplementary Material


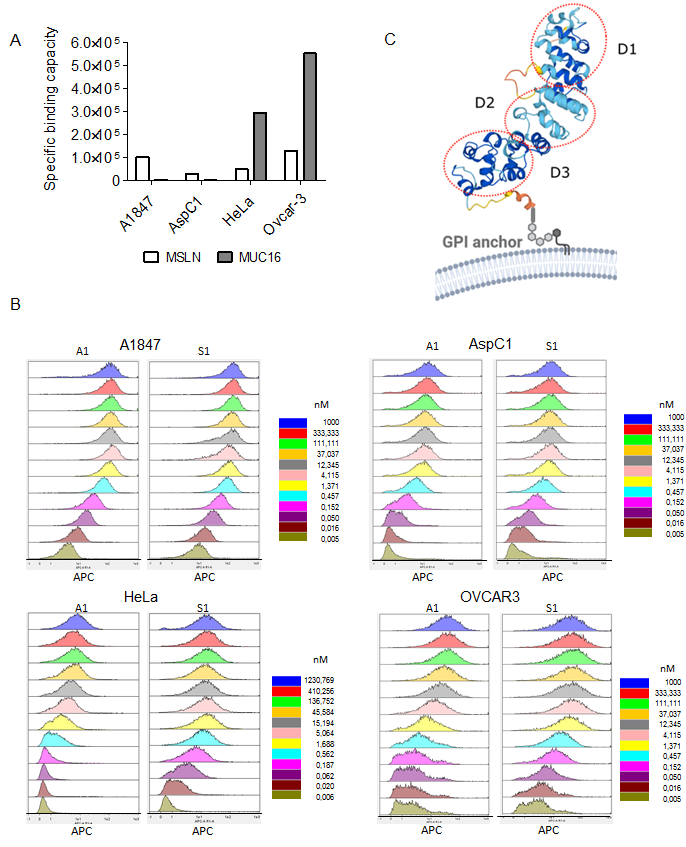


**Supplementary Figure 1**

A) Quantitative determination of MSLN and MUC16 surface expression on cancer cell lines using Qifikit (Agilent). B) Histogrammes of Nb binding on tumor cells. C) Modelisation of human mesothelin (34,35) (AlphaFold Protein).


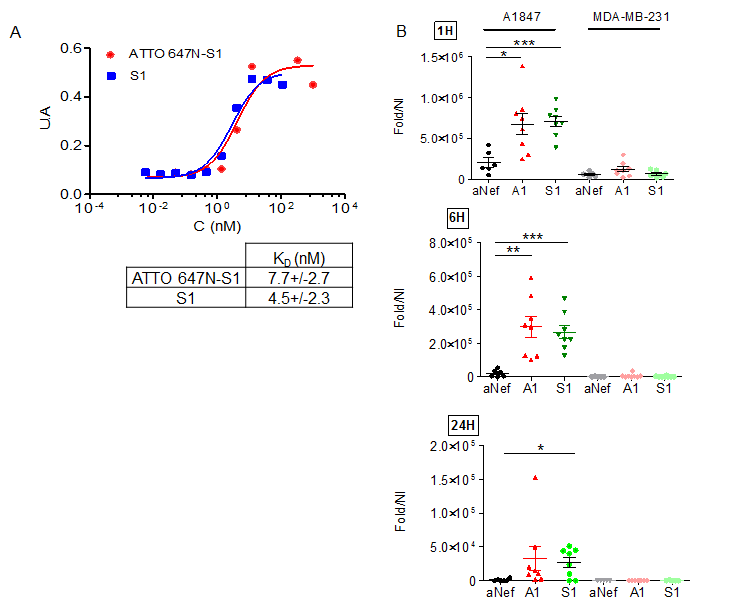


**Supplementary Figure 2**

A) Representative binding curve of ATTO 647N-S1 and his-cmyc-tagged S1 on immobilized recombinant MSLN. Binding was detected with a mouse HRP-conjugated anti-HIS mAb. B) Quantification of tumor fluorescence intensity in whole body 1h, 6h and 24h post-injection. Fluorescence intensity of the negative ROI was substracted to the fluorescence intensity measured in the tumor ROIs and expressed as fold change compared to fluorescence in ROI in non injected mice (n=5-8 mice/group). The p-values were calculated with two-tailed unpaired t-test, * p<0.05, ** p-value < 0.01, *** p-value<0.001.


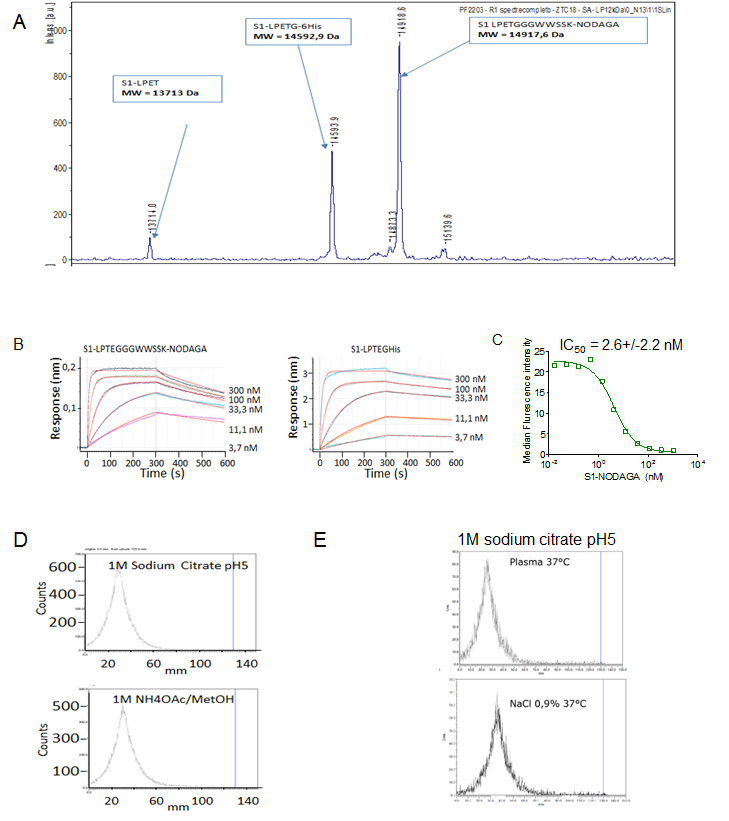


**Supplementary Figure 3**

A) Representative MALDI-TOF MS profile of NODAGA-S1 after purification by size exclusion chromatography. B) Binding kinetic parameters of sortag-S1 and NODAGA-S1 on recombinant MSLN determined by bio-layer interferometry, using biotinylated MSLN immobilized on streptavidin-tips (BLI). Association and dissociation constants were determined by fitting the curves to a 1:1 interaction model . C) Binding capacity of NODAGA-conjugated Nb S1 on A1847 cells by competition experiment with S1. Serial concentrations of NODAGA-S1 were incubated with A1847 cells in the presence of Nb S1. Curves were analyzed using the one site total binding. D) Radiochemical purity : representative profiles of thin layer chromatography after ^68^Ga radiolabeling of NODAGA-S1. Upper panel : migration in 1M Sodium Citrate pH 5.0 to detect free gallium-68 and [^68^Ga]Ga-NODAGA peptide; lower panel : migration in 1M NH4OAc/MetOH ((1/1, v/v) to detect free [^68^Ga]Ga-NODAGA peptide. E) Representative profiles of thin layer chromatography of [^68^Ga]Ga-NODAGA-S1 after 2h incubation at 37°C in human plasma or NaCl 0.9%, in 1M sodium citrate pH 5,0.
